# A Technic for Constructing Artificial Dentures*Read before the National Dental Association at its twenty-second annual session, Chicago, Ill., August 5-9, 1918, and published in full in the *Journal of the National Dental Association*, December, 1918.

**Published:** 1919-01

**Authors:** M. M. House


### A TECHNIC FOR CONSTRUCTING ARTIFICIAL DENTURES *

BY M. M. HOUSE, D. D. S.

The mastering of a technic simply means habituating one's self to do a certain thing in a certain way; it means a clear and definite plan of what to do and how to do it and makes for the easier and surer formation of the habit. Such habits are the only ones to be depended upon in the emergency of our daily practice.

With this thought in view I present the following technic based upon the necessity for the greatest efficiency in artificial dentures. This necessity is illustrated by the "experiments of Dr. G. V. Black, begun in 1893, for the purpose of showing the pressure that it was possible to exert on the human teeth, and which brought out the fact in a tabulation of results that in one thousand persons, the average force exerted was 171 pounds on the molar teeth but much less on bicuspids and incisors. The variation was from 25 to 275 pounds," whereas the greatest force it was possible to exert on the same machine where artificial dentures were worn was from zero to 50 pounds. "Force required in the mastication of meats showed that the most tender meats required 3 to 5 pounds pressure, while tougher meats required from 15 to 90 pounds."

While these experiments were made by Dr. Black for the purpose of determining the resistance form required in cavity preparation, they have an equal application in determining the masticating limitations we may expect in artificial dentures, and suggest the need of a deeper cusp arrangement. This in my opinion, unquestionably allows of a greater masticating efficiency, which after all is the

* Read before the National Dental Association at its twenty-second annual session, Chicago, Ill., August 5-9, 1918, and published in full in the *Journal of the National Dental Association*, December, 1918.

most essential function of the teeth, whether natural or artificial.

To begin with, it is absolutely necessary to make a careful mouth examination, noting with care the tissue conditions, muscle attachments and movements, as well as the amount of absorption and the loss of facial contour. This examination is indicated as essential because the peripheral border of the impression is the first step in the restoration of said lost facial contour, the restoration of which is the patient's chief idea of a successful result and the attainment of which is usually his chief concern, though but one of the features to be considered by a really conscientious dentist.

The first step in the impression is the fitting of a metal tray which closely approximates the area to be covered, care being taken that the borders at no point are high enough to raise the soft tissues or muscle attachments. An impression is now taken with S. S. White Tray Compound in the same way that any ordinary modelling compound impression would be taken after the method of Dr. Rupert E. Hall.

This tray compound is then removed from the metal tray, and trimmed to approximate proper facial restoration, care being taken to lower the margins so there will be no impingement on the muscle attachments and no displacement of tissue around the peripheral border.

Referring in particular to the lower impression it is necessary to have the buccal margins of the compound tray trimmed, so there is no impingement of the muscles in the masseter region, nor overlapping of the cheek tissues.

In the lingual region it is necessary to make a very careful examination with the finger, the patient's tongue being raised to the roof of the mouth, thus plainly allowing the points of attachment of tissue to the bone along the floor of the mouth, to be felt.

If in doubt as to one's ability to properly trim the lingual margin of the lower tray, so there will be no impingement of tissue and yet retain all the lingual flange applicable to the case in hand, a simple way to get a perfect margin in this region is to trim the compound tray slightly shorter than the required length and trace on this margin with Kerr's modelling compound, a small area at a time, then slightly heat over a fine point flame, dip in warm but not hot water, place the tray in the mouth and hold it down with the forefingers, having the patient raise the tongue to the roof of mouth, moving it freely in all directions. When the entire lingual border has been traced in this manner and thoroughly chilled, first trim away all undercuts, especially in the region of the mylohyoid ridge, then trim the edge with a knife to the thickness desired for the finished denture.

In the buccal and labial region, the compound tray should be trimmed to about the thickness of a britannia metal tray. This affords a free and unrestrained outlet for the flow of plaster, allowing no bulky masses to accumulate so that in the proper massaging of the cheeks and lip, it is possible to obtain the desired contour of these regions. In all cases after trimming, the tray should be placed in the mouth and a test made for impingement of muscle attachments and soft tissues.

For successful results, certain qualities are essential in the plaster to be used in these impressions. Owing to the small amount of plaster which it is possible to use in these impressions, it must have peculiar qualities of hardness, smoothness, a uniform working property and a not too rapid setting.

The time required for the setting is governed by two things: the temperature of the water and some chemical agent, the amount of which must be determined by the operator. Salt is the agent commonly used but personally, I much prefer potassium sulphate as it does not, in any way, deteriorate the quality of the plaster when set.

To mix it, use tepid water with a small amount of potassium sulphate well dissolved, then sift the plaster, allowing it to become saturated as it settles in the solution. After a sufficient amount has been sifted, pour off the excessive water and stir thoroughly. You will now have plaster of a thin creamy consistency, from which, by waiting for it to slightly set, you may get any consistency you desire for the special case in hand, as the tissue condition of each mouth requires a special treatment in order to obtain the desired result, though the plaster must never be sufficiently stiff to be resistable in moulding the peripheral borders. I speak of moulding in this connection, because I employ a method of drawing the attachments involved to their low point of attachment and then massaging the face along the peripheral borders of the impression to mould them to proper form.

Note particularly at this point the fact that the upper and lower plaster impressions are taken in totally different manners.

Let us first consider the upper impression. The upper compound tray being properly trimmed for facial restorations and lowered for muscle and tissue attachments, the undercuts sufficiently trimmed to permit the plaster to flow freely past, we now place a sufficient amount of plaster mixed to proper creamy consistency in the tray (the patient having been previously instructed to completely relax the facial muscles), place the tray in the mouth and move to place with gentle wavelike oscillating guidance, until seated by the test of gentle touch. When the plaster is of the proper consistency, mould the borders by holding the tray in place with the forefinger of each hand and with the thumbs, draw the lip in region of the phrenum and the facial muscles in the region of muscle attachments, freely downward. Then, with the thumbs, massage the facial tissues gently along the peripheral borders of the impression to properly muscle trim and perfect the facial contour as well as to properly place the soft marginal tissues. After the plaster is sufficiently set, remove and examine it to make sure that you have an ideal impression of the case. In case of failure, either remove all the plaster from the compound tray and retake the impression or trim the plaster properly (trimming out well in the muscle and phrenum regions), then soak it thoroughly in water, placing in it a small amount of creamy plaster and replace in the mouth, using the same technic for placing tissues as before. This description covers the general technic used for this work with the exception of post damming, though, of course, it is impossible to consider here the many variations with which we have to deal in different cases.

In order to post-dam the upper impression thoroughly dry the plaster along the posterior border with cold compressed air, then, with a stick of Kerr's Modelling Compound, trace a small area across the posterior border up to the point of the hard palate, heat over a fine point flame; dip quickly in warm water, place in the mouth and, with firm and steady pressure, gently seat the impression in place. Remove and trim to the width and shape desired for the case in hand and if not satisfactory, re-heat and proceed as before until the desired result is obtained.

### LOWER IMPRESSION

Having your compound tray fitted, trimmed and tested, making sure it does not impinge on any of the movable tissues, and with undercuts sufficiently removed to allow the plaster to flow freely by, proceed with the mixing of the plaster as for the upper impression. Having the patient with facial tissues entirely relaxed, place the compound tray with plaster of the proper consistency in the mouth and have the patient place his tongue in a high and forward position. Then gently move the tray to a seated position as before. Have the patient keep his tongue in the position noted above, hold the tray in place with the forefinger of each hand about the region of the first molar, then with the thumb and second finger of each hand, draw the facial tissues upward and inward over the outside borders, to properly muscle trim and perfect facial contour as well as to place the soft marginal tissues. When the plaster is sufficiently hard, remove and examine it carefully. In event of failure, before removing the plaster from the tray, correct any defects in the compound tray which may have caused impingement or displacement of tissue. Always remove all the plaster in the lower tray in case of failure, before taking another impression.

After the ideal impression is attained by this method, you will have a perfectly trimmed outer border with the excess plaster drawn up over the top of the tray, but the excess of plaster around the inner border will be spread in thick rolls between the tongue and the tray, leaving the margins much thicker than desired in the finished plate. It is, therefore, necessary to trim away this excess until it represents the thickness you desire to reproduce in the finished denture, remembering that this border would be represented by a groove in the cast for reproduction of this margin of the finished plate.

Models for vulcanizing on are made of Weinstein's Artificial Stone, or Spence's plaster to insure adequate strength. The models should be cast in a form that will insure a reproduction of the peripheral borders of the impression and give proper form and size, without excessive trimming, to permit flasking and give the greatest possible strength in the models.

The bite plates should be made of Ash's Soft Tray Metal, and should be swaged to accurate adaptation on the models. The wax for the bites is then placed on the plates in accordance with a form gage to secure coaptation, and the waxes are trimmed so that the occlusal contact is hardest in the region of the last molars, while the lip lengths are determined by carving the wax or otherwise indicating them on the waxes. This results when the bites are replaced in the mouth in actual contact in the molar regions with a space of three-sixteenths of an inch in the incisal region. The lower wax bite is then removed from the mouth and a groove of soft wax is made in it with a hot spatula from near its distal extremity to the incisors on both sides. Care should be taken to melt the wax only in the center, leaving a thin wall of harder wax all around the margins to hold the softened wax in the groove. Dr. House calls this pooling the wax in the groove. The idea is to make this wax so soft that it will be easily compressed when the waxed lower bite is returned to the mouth and the patient makes an occlusal bite.

The lower bite plate is now placed in the mouth and the patient instructed to bring the lips together, thus causing the bite plates to come together in their proper relation, the hard wax left in the region of the lower incisors automatically stopping the jaws at the proper rest point. It is safe to assert that the jaws will never be too close together if this method is used; it does not confuse the patient, eliminates the liability of excessive pressure on either side of the mouth, thus removing one of the most common faults in our procedure of construction.

It is now necessary to examine the patient's profile, general contour of the face, and in particular, the appearance of the lips at rest. Proper facial contour should be established at this point and if advisable to close the jaws, to get the proper appearance of the lips, all that is necessary to accomplish this, is to remove the lower bite plate from the mouth and cut a notch out of the wax from lateral incisor to lateral incisor inclusive, and to a depth from 1-32d to 1-16th of an inch of whatever is deemed sufficient by the operator, to give a proper length of bite, or the working bite.

Re-pool the wax and proceed as before, then mark the median line and carefully test the bite.

This procedure first described by Dr. Greene in his method of taking bites (although he used modelling compound instead of wax), gives the operator absolute and scientific control as to lip rest line and proper facial length.

In my experience this method, used in connection with the jig designed by Dr. Hall for mounting cases in his articulator (according to the Bonwill measurements of the equilateral triangle) obviates the use of the face bow.

### MOUNTING OF CASTS

These casts are mounted in the Hall articulator according to Dr. Hall's method, by setting the center pin of the jig at the median line and occlusal plane and the posterior pins in line with the occlusal plane.

### WAXING UP TEETH

This is a feature of the work which must be accurately and carefully carried out for with the facial contour properly re-established with bite plates, we are enabled to place the teeth so as to obtain the best effect,—that of naturalness.

The thickness of the upper plate is obtained by the use of the 20 guage metal used in the bite plate with an additional thickness of Kerr's Cast Gold Wax, 28 guage; a second thickness of the same wax is used where metal relief is indicated for hard tissues. A No. 60 tinfoil perfectly conforming to this surface of the plate is then burnished over the palatal portion, leaving the edges higher than the occlusal surfaces of the teeth to engage in the plaster when invested.

The case is then invested and when packed, a relief of the hard areas on the cast is made with No. 60 tinfoil and the entire surface of the cast covered with a very thin tinfoil made for this purpose. The tin covered cast is then soaped, the flask closed and the case vulcanized.

By this method the greatest density and strength and the utmost elasticity of rubber is obtained, as it is vulcanized between metals and uniform palatal thickness made throughout, thus eliminating the danger of warping in polishing, as both palatal surfaces of the plate come from the vulcanizer practically finished. Of inestimable advantage to the patient, is the fact that in this treatment of the rubber, nature is so closely simulated that the least irregularity of surface is reproduced and the patient relieved from the discomfort of the ordinary thick, smooth palate.

### TAKING TEST BITE FOR RE-GRINDING AND BALANCING OF DENTURES

Taking the final test bite with finished dentures in the mouth, remounting on anatomical articulator and grinding to all the movements of the mouth with carborundum paste, make it possible to obtain a degree of accuracy and efficiency in occlusion and articulation never hitherto accomplished. This method, in connection with the incisal guide pin and cup as advocated by Dr. Hall, has made possible the advanced and unheard-of efficiency of the deep cusp teeth.

We may readily see how slow we have been to receive and perfect the good things offered us by early geniuses in our profession by referring to the treatment of this subject by Dr. Greene, whose book was published in 1910. This was years after the invention of his novel scheme for re-cutting the occlusal surfaces of the teeth by mounting the finished dentures in the articulator and working them together, with carborundum powder placed between the teeth, for the purpose of cutting out the bumps and inaccuracies.

### TEMPORARY PLATES AND DUPLICATION

In closing, let me call attention to a most satisfactory technic employed for reproduction, where cases present for full dentures before the extraction of the teeth has been accomplished. A plaster impression is taken of the mouth prior to extraction, a record made of the patient's name, address, telephone number and the shade of the natural teeth; both impression and record are filed. After extraction, the teeth are boiled and cleaned, placed in the plaster impression and a plaster cast made which retains the teeth in their original position. These casts are also filed and kept for the purpose of duplicating the tooth form, arch form, fillings, crowns and irregularities and are helpful aids in the reproduction of grooves and pitted surfaces, as well as for staining and shading the porcelain teeth for the denture to be made. They thus serve as guides for the building of both temporary and permanent dentures.

A surgical procedure is employed in the preparation of the mouth after extraction as originated by Dr. Carl D. Lucas, of Indianapolis, and presented by him in a paper before the First District Dental Society of Michigan and later published in the Michigan Dental Journal in 1916.

This procedure makes it possible for people to wear temporary plates with great comfort and satisfaction, after six or eight days from the time of extraction, and their mouths are then as smooth and free from prominent points and undercuts, as they would be without this surgical preparation in four or five months.

In cases of people in public life who would be greatly inconvenienced by being without teeth for even this length of time, the following procedure is used: all of the teeth posterior to the cuspids are removed and the mouth surgically treated and allowed to heal for a week or ten days. Impressions and bites are then taken as for partial plates, the cases articulated and the teeth selected and ground to duplication of form. The upper central is then sawed out of the cast and the cast carved, so as to permit the artificial tooth to be set in the same alignment and position as the natural tooth. Working from the center in each direction, all the teeth are treated in the same manner, both above and below, making complete artificial dentures. After these dentures are vulcanized and finished, the patient is called, the remaining natural teeth extracted and the mouth surgically treated. The dentures are then fitted to place and the patient leaves with dentures comfortable to wear while the healing is being effected.

In all upper cases of this kind we use a large size Eureka Suction as no anterior gum is put in these cases at this time.

The patient is instructed to return in six or eight days for the removal of the sutures, at which time, with the denture held firmly in place, an impression is taken of the anterior portion of the mouth where the gum was omitted. The impression and denture are then removed, the Eureka rubber taken off and a cast is poured into the denture. A front is waxed in, flasked, packed, vulcanized and finished. The stud for the Eureka is then cut off and the temporary plate is complete.

The same technic is employed in the lower case with the omission of the Eureka.

I have found this method very successful and highly satisfactory to the class of business and professional people referred to above.

